# Association of Amoebae and Dysentery Bacilli

**Published:** 1916-10

**Authors:** 


**Association of Amoebae and Dysentery Bacilli.** Rousel, Brule and Barat, Soc. Med. des Hop., Feb. 25, believe that this is due to simultaneous infection, the amoebic lesions predisposing to bacillary infection. While bacillary dysentery is common in campaigns, especially in the tropics, and is thus easily introduced into armies in other climates, they emphasize the importance, even when the bacilli have been demonstrated, of seeking for amoebae.

